# Wind Dynamic Characteristics and Wind Tunnel Simulation of Subgrade Sand Hazard in the Shannan Wide Valley of the Sichuan–Tibet Railway

**DOI:** 10.3390/ijerph19148341

**Published:** 2022-07-08

**Authors:** Shengbo Xie, Xian Zhang, Yingjun Pang

**Affiliations:** 1 Key Laboratory of Desert and Desertification, Northwest Institute of Eco–Environment and Resources, Chinese Academy of Sciences, Lanzhou 730000, China; zhangxian@nieer.ac.cn; 2 University of Chinese Academy of Sciences, Beijing 100049, China; 3 Institute of Desertification Studies, Chinese Academy of Forestry, Beijing 100091, China; pangyingjun@caf.ac.cn

**Keywords:** wind dynamic, railway, subgrade, wind tunnel simulation, Sichuan–Tibet Railway

## Abstract

The Shannan wide valley section of the Sichuan–Tibet Railway is located in the middle reaches of the Yarlung Zangbo River, where sand hazard is severe. A wind tunnel simulation experiment was conducted by establishing a subgrade model and performing field observation to carry out research on the dynamic environment of blown sand and the sand hazard formation mechanism of subgrade in the Shannan wide valley. Observation results showed that the sand-moving wind of the Shannan wide valley was chiefly derived from the ENE direction, and the resultant sand transport direction was WSW. Wind speed, the frequency of sand-moving wind, the sand drift potential, and the maximum possible sand transport quantity were relatively high in the spring. Meanwhile, the dynamic of the wind-blown sand flow was further enhanced in the spring, particularly influenced by the flow action of the Yarlung Zangbo River. Thus, sand hazard mainly occurred in the spring. The Sichuan–Tibet Railway subgrade evidently changed the wind speed, the wind-blown sand flow field, and conditions of transport and accumulation. Within the distance of 5 times the model height in the windward direction and at the subgrade top center to 20 times the model height of the leeward direction was the wind speed deceleration zone, resulting in sand particle sediments. A wind speed acceleration zone appeared on the subgrade windward slope shoulder, resulting in wind-blown sand flow erosion. This study provides a scientific basis for sand hazard prevention and control in the Sichuan–Tibet Railway.

## 1. Introduction

**Figure 1:**
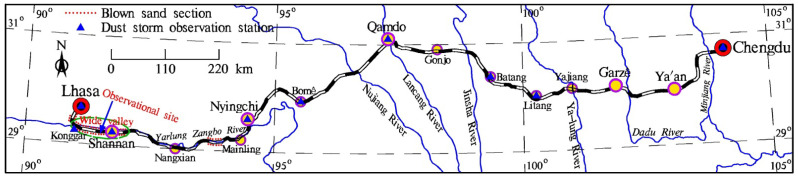
Schematic map of the Shannan wide valley and the observation stations along Sichuan–Tibet Railway.

The Sichuan–Tibet Railway is an east–west high-speed railway linking Sichuan Province with the Tibet Autonomous Region (TAR). This railway starts from Chengdu, the capital city of Sichuan Province, in the east; runs through Ya’an, Garzê, Qamdo, Nyingchi, and Shannan; and ends in Lhasa, the capital city of TAR, in the west, with a total length of about 1550 km (Figure 1). It is the second “Sky Road” into Tibet after the Qinghai–Tibet Railway and one of the trunk railways in Southwest China. The Sichuan–Tibet Railway line is characterized by extreme cold and hypoxia, complex terrain, criss-crossing mountains and valleys, diverse climate, unpredictable weather, fragile ecological environment, and frequent natural disasters. Blown sand is one of the main natural disasters along the Sichuan–Tibet Railway and is concentrated in the Shannan wide valley section in the middle reaches of the Yarlung Zangbo River (Figure 1). Affected by local factors such as dry and windy climate, abundant sources of sand, sparse and low vegetation, a short growing season, and intensified human activities, land desertification is severe in the Shannan wide valley [1,2,3], which has typical aeolian geomorphology [4,5] and considered the most critical area in the Yarlung Zangbo River basin and even the entire Qinghai–Tibet Plateau. Large areas of moving dunes and sandy land are accumulated at the valley bottom, valley slopes, and narrow mountain passes [6,7,8] and distributed in discontinuous bands (patch and flaky), with strong blown sand activities, thereby substantially endangering the railway. Owing to the unique alpine blown sand environment of the Qinghai–Tibet Plateau [9,10,11], any slight disturbance will change the material and energy balance of the system [12]. During railway construction, the originally sparse vegetation and fragile ecology along the line will be destroyed inevitably [13]. In particular, the appearance of railway subgrade disturbs the original relatively stable dynamic balance of blown sand activities on the plateau [14], changes the running path and intensity of near-surface blown sand flow [15,16,17], and aggravates the blown sand hazards. Survey statistics have indicated that approximately 10 km of railway lines in the Shannan wide valley of the Sichuan–Tibet Railway have blown sand hazards (Figure 1), which present the most concentrated blown sand distribution and are the most dangerous hazards along this railway. At present, relevant research mainly focuses on the railway track, such as wall barrier properties along the railway track [18], optimization of a railway sanding system for optimal grain entrainment into the wheel-rail contact [19], reducing sand deposition on railway tracks to improve transportation [20], wind-blown sand action on civil structures [21], and railway track maintenance in sandy areas [22]. Few studies have been performed on the wind-blown sand hazards of railway subgrades, particularly in the wind–sand area of south Tibet valley with high elevation and cold temperature. The wind dynamic environment of the Shannan wide valley section of the Sichuan–Tibet Railway remains unclear, and the sand hazard formation mechanism of the subgrade has not been reported. These aspects are conducive to taking measures for targeted sand prevention. Therefore, this study aims to provide a scientific basis for the prevention and control of wind-blown sand hazard along the Sichuan–Tibet Railway and have reference significance for the prevention and control of wind-blown sand hazards in railway subgrades in other sandy areas.

## 2. Research Methods

### 2.1. Field Observation

**Figure 2:**
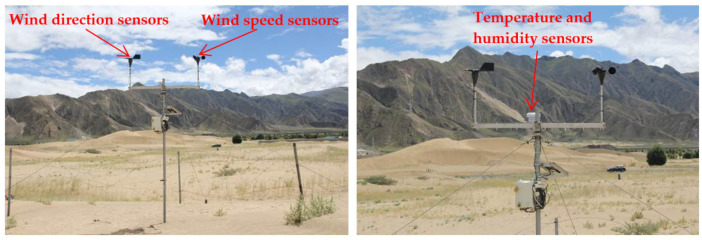
Wind-blown sand observation in the Shannan wide valley of the Sichuan–Tibet Railway.

The Shannan wide valley section of the Sichuan–Tibet Railway, which is located in the middle reaches of the Yarlung Zangbo River, is an alpine valley sand area south of Tibet. The route form is mainly roadbed, and the main landform along the line is an east–west valley. Distributed in the valley are barchan dunes, exposed sandy land, gobi, climbing dunes, and shadow dunes [23]. The geographic coordinates of the observation site are 29°15′39″ N, 91°31′14″ E, with an altitude of 3562 m. Meteorological sensors present a surface height of 2 m, with wind speed, direction, temperature, and humidity recorded every 5 min (Figure 2). The observation period was from November 2019 to October 2020. First, the collected observation data were used to analyze the average wind speed and sand-moving wind conditions statistically. Second, the sand drift potential (DP) was calculated in accordance with the method proposed by Fryberger and Dean [24], as shown as follows:

(1) $$DP=V^{2}(V-V_{t})t$$

where the DP is the sand drift potential expressed in vector units (VUs); V is the wind speed higher than the sand-moving wind (m·s^−1^); V_t_ is the sand-moving wind speed, namely, the critical starting wind speed of sand particle (m·s^−1^); and t is the time affected by the sand-moving wind and is expressed in frequency. The resultant drift potential (RDP) and resultant drift direction (RDD) were calculated by synthesizing the sand DP on the basis of the vector synthesis rule, and the index of directional wind variability was computed as the ratio of the RDP to the DP (RDP/DP). Third, the maximum possible sand transport quantity (Q) was calculated in accordance with the method proposed by Ling [25], as shown below:

(2) $$Q=8.95\times 10^{-1}(V-V_{t})\times T$$

where Q is the maximum possible sand transport quantity (kg·m^−1^·a^−1^); V is the wind speed greater than the sand-moving wind (m·s^−1^); V_t_ is the sand-moving wind speed, namely, the critical starting wind speed of sand particle (m·s^−1^); and T is the cumulative duration of wind speed with different ranges. The resultant quantity (RQ) and resultant angle (RA) of the maximum possible sand transport were calculated by synthesizing the Q on the basis of the vector synthesis rule.

The critical starting wind speed of sand particle was measured on site, a portable wind-measuring device was installed on the sand surface, and the wind speed was increased slowly. At the same time, the initial movement of sand particle was monitored using a high-speed camera. The critical starting wind speed of sand particle was 5.0 m·s^−1^. Aeolian sand flow is a two-phase flow [26]; there are three basic forms of sand movement: surface creep, saltation movement, and suspension movement. Among them, saltation movement is the most important form of aeolian sand movement, and the saltation sand materials account for more than half or even 3/4 of the total amount of sand in aeolian sand flow. The aeolian sand movement of the Shannan wide valley section of the Sichuan–Tibet Railway is also dominated by saltation movement, which is consistent with this law. The sand materials swing back and forth in the valley by saltation movement.

### 2.2. Wind Tunnel Simulation Experiment

**Figure 3:**
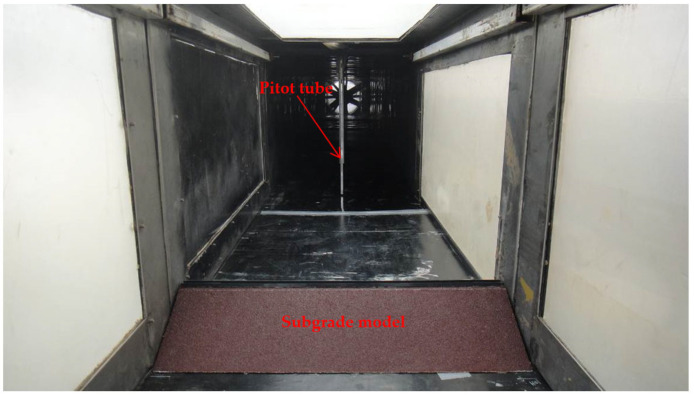
Subgrade model for the wind tunnel experiment.

A wind tunnel experiment was conducted in a wind tunnel of the Key Laboratory of Desert and Desertification, Chinese Academy of Sciences. The direct current closed-mouth blowing wind tunnel has a length of 6 m and a cross section of 0.63 × 0.63 m^2^. The boundary layer thickness at the bottom is 12–15 cm. The wind velocity in the wind tunnel can be continuously adjusted in the range of 0–20 m·s^−1^. The Sichuan–Tibet Railway is I heavy rail, the subgrade top width is 3.5 m, and the slope ratio is 1:1.75 (i.e., slope of 30°) according to the specifications of railway subgrade design (TB10001-2016, National Railway Administration of the People’s Republic of China, Beijing, China). The subgrade model size of the wind tunnel experiment had a similarity ratio of 1:100. The model was 8 cm high and 62 cm long, and it was pasted with 36-mesh sandpapers to simulate the surface roughness of the real field subgrade (Figure 3).

**Figure 4:**
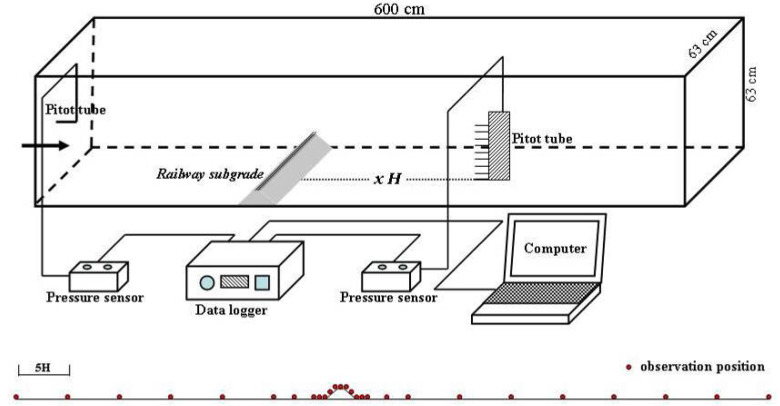
Layout of the subgrade model for the wind tunnel experiment and schematic of each observation position.

**Figure 5:**
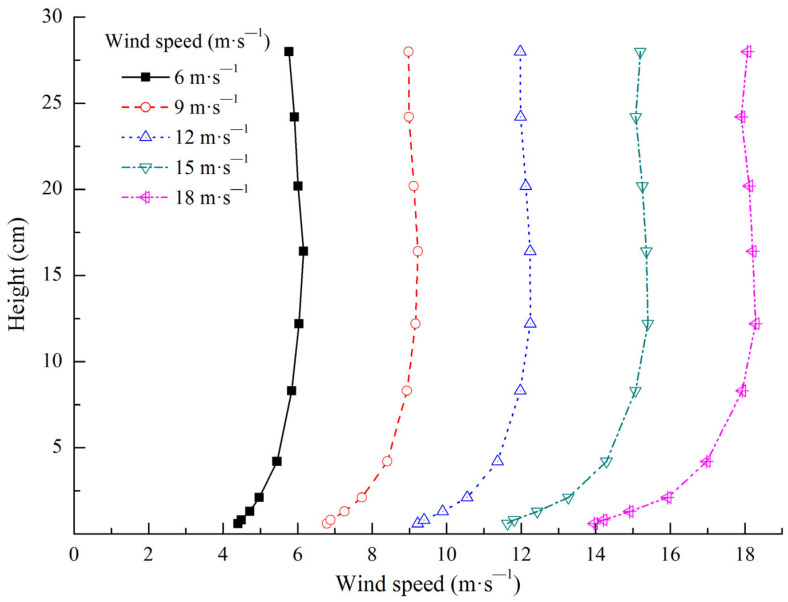
Wind speed profile of the wind tunnel experiment.

The 10 measurement positions were −30H, −25H, −20H, −15H, −10H, −5H, −3H, −1H, −0.5H, and −0H on the subgrade windward side (H represents the model height of the subgrade, − represents the windward side, + represents the leeward side, and −0H represents the windward subgrade slope foot, the same below). The five observation positions were set on the subgrade surface, middle windward slope, windward slope shoulder, subgrade top center, leeward slope shoulder, and middle leeward slope. The 12 measurement positions were 0H (representing the leeward slope foot, the same below), 0.5H, 1H, 3H, 5H, 10H, 15H, 20H, 25H, 30H, 35H, and 40H on the subgrade leeward side (Figure 4). The 10 different heights of wind speed at each position were converted using pitot tubes, which were placed at the bottom center of the wind tunnel. The measured wind speed heights were 0.6, 0.8, 1.3, 2.1, 8.3, 12.2, 16.4, 20.2, 24.2, and 28 cm to observe the entire boundary layer of the experimental section without exceeding the central height, thus avoiding interference from the top of the wind tunnel and its boundary layer. After the experimental wind speed stabilized, it was measured every 2 s for 30 consecutive times, and the average value was taken. The sand-moving wind speed in the Shannan wide valley is 5 m·s^−1^, According to the local wind sand observation results, the instantaneous maximum wind speed is 21.64 m·s^−1^; namely, the wind speed range of local blown sand activities is 5–21.64 m·s^−1^. Therefore, five experimental wind speeds were selected to conform to the local actual situation (i.e., 6, 9, 12, 15, and 18 m·s^−1^). The wind speed profile of wind tunnel experiments is shown in Figure 5. The wind speed was stable at above 8.3 cm height, which conformed to the logarithmic distribution law of wind speed with height on a uniform bed when the height was below 8.3 cm. Relevant research has shown that the greater the angle between the railway and the main wind direction is, the more serious blown sand hazards will be [27,28]. With reference to relevant research [29,30,31], the angle between the subgrade model and the wind direction was set to 90°.

The wind tunnel experiment conformed to the principles of wind kinematic similarity and dynamic similarity. This study ensured that all measurement positions were located within the wind tunnel test section by moving the pitot tube and the railway subgrade model. Given that a rectification section existed at the upwind direction of the wind tunnel test section, the stability of wind speed and direction in the test section could be ensured by rectifier rectification. Therefore, the wind conditions in the wind tunnel test section were very similar to those in nature, which conformed to the principles of wind kinematic similarity and dynamic similarity. The measured initial wind speed profile in the wind tunnel test section conformed to the logarithmic distribution law of wind speed with height (below 8.3 cm height) in nature (Figure 5), which also illustrated this point.

## 3. Results

### 3.1. Dynamic Wind Environment

**Figure 6:**
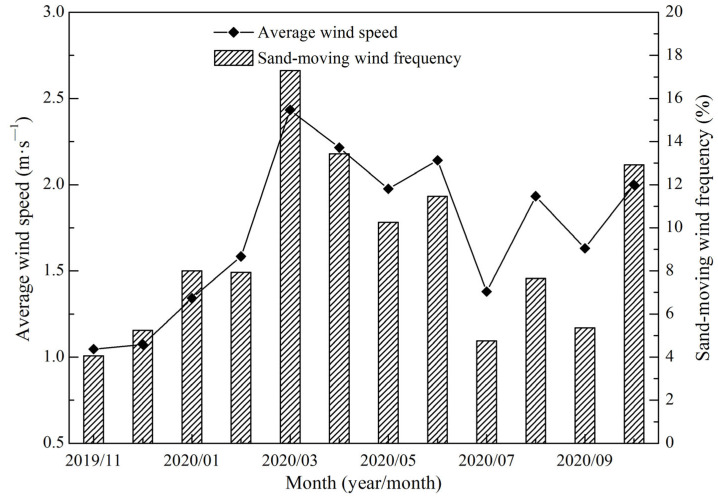
Monthly variations in the average wind speed and sand-moving wind frequency in the Shannan wide valley of the Sichuan–Tibet Railway.

**Figure 7:**
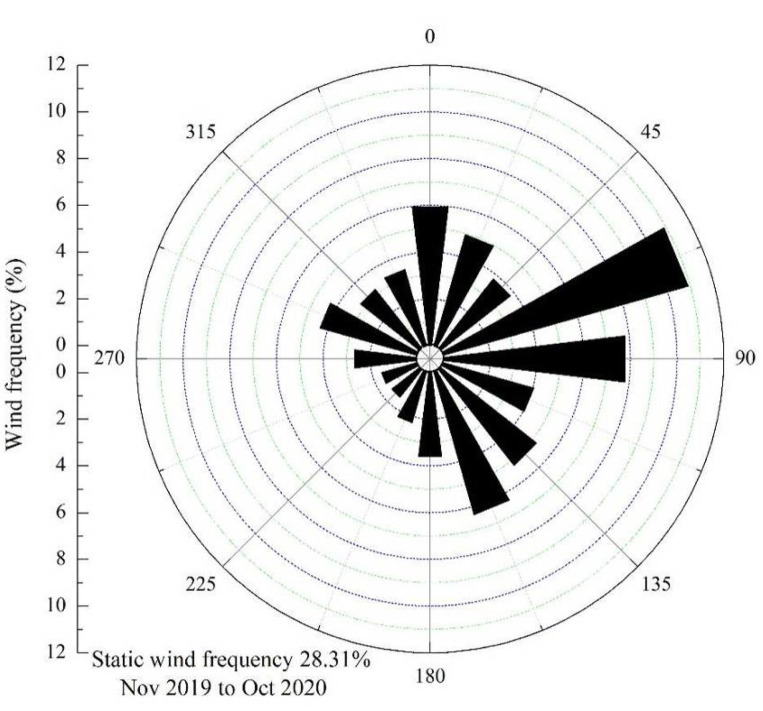
Rose diagram of the annual wind in the Shannan wide valley of the Sichuan–Tibet Railway.

The annual average wind speed in the Shannan wide valley was 1.73 m·s^−1^, the instantaneous maximum wind speed was 21.64 m·s^−1^, and the maximum 5-min average wind speed was 13.09 m·s^−1^. The average wind speed was high during the spring and reached a maximum in March at 2.43 m·s^−1^. In contrast, the average wind speed was low during the winter and at minimum in November at 1.05 m·s^−1^. The average wind velocities during the summer and autumn were between those in the aforementioned two seasons (Figure 6). From the annual wind rose data, the ENE wind direction was prioritized, accounting for 10.91% of the annual total; followed by the wind direction of E, which accounted for 7.82% of the annual total. The static wind frequency was 28.31% (Figure 7). The annual frequency of sand-moving wind was 9.05%. A high frequency was recorded in the spring and highest in March at 17.31%. In contrast, a low frequency was recorded in the winter and lowest in November at only 4.05%. The frequency of sand-moving wind in the summer and autumn was between 4.05% and 17.31%. The monthly variations in sand-moving wind frequency and average wind speed were consistent (Figure 6).

**Figure 8:**
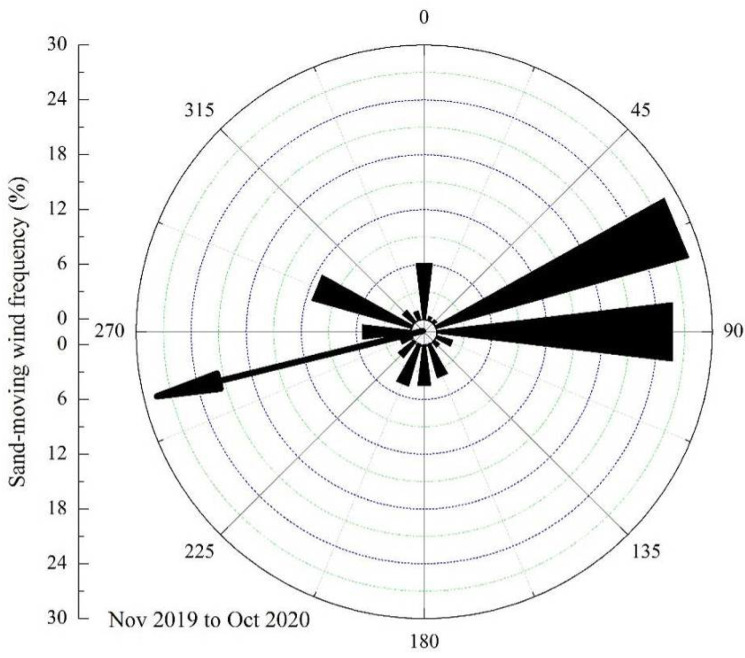
Rose diagram of the annual sand-moving wind in the Shannan wide valley of the Sichuan–Tibet Railway.

From the annual sand-moving wind rose data, the wind direction of ENE was given priority, followed by the E wind direction, accounting for 28.57% and 25.83%, respectively, of the annual total. The resultant direction of sand-moving wind was 76.33°, which was an ENE direction (Figure 8).

**Figure 9:**
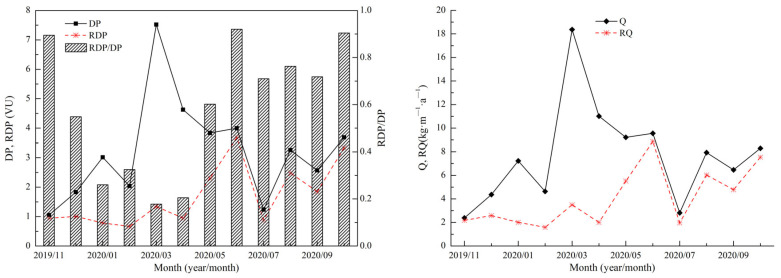
Monthly variations in the DP and the Q in the Shannan wide valley of the Sichuan–Tibet Railway.

The sand DP and the Q were high in the spring (March–May) and low in other seasons (Figure 9). In addition, the resultant sand transport direction (including the RDD and RA of the maximum possible sand transport) was dispersed (Table 1).

**Figure 10:**
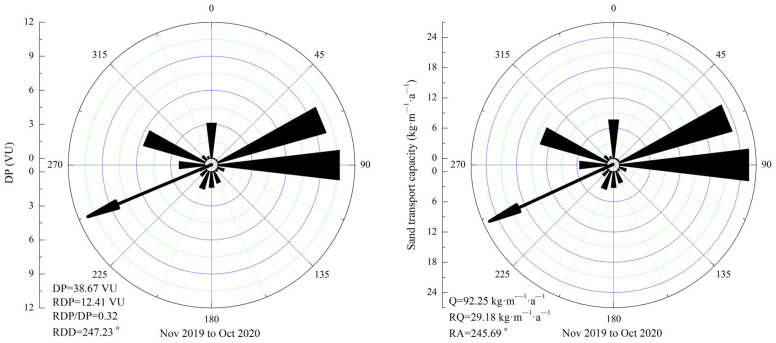
Rose diagram of the annual DP and the maximum possible sand transport quantity in the Shannan wide valley of the Sichuan–Tibet Railway.

The annual DP of the Shannan wide valley was 38.67 VU. The annual RDP was 12.41 VU, and the annual direction variability index (RDP/DP) was 0.32. The annual RDD was 247.23°, which was a WSW direction (Figure 10). The annual Q was 92.25 kg·m^−1^·a^−1^, and the distribution of the greatest contributing wind speed ranged from 7 to 8 m·s^−1^ (Table 2). In addition, the annual RQ was 29.18 kg·m^−1^·a^−1^, and the annual RA was 245.69°, which was a WSW direction (Figure 10).

### 3.2. Formation Rule of Sand Hazard

**Figure 11:**
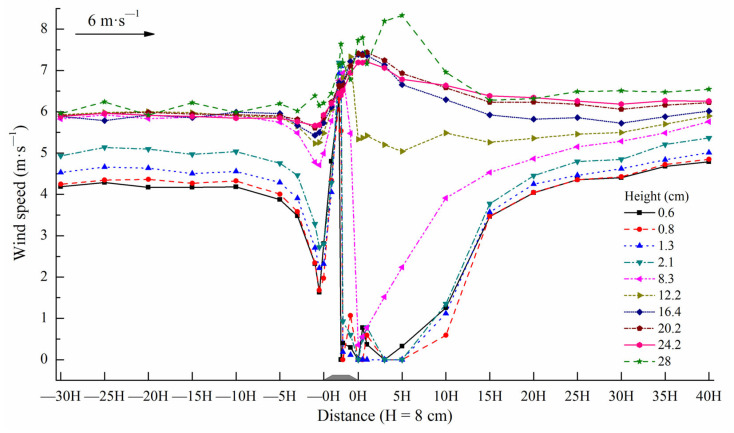

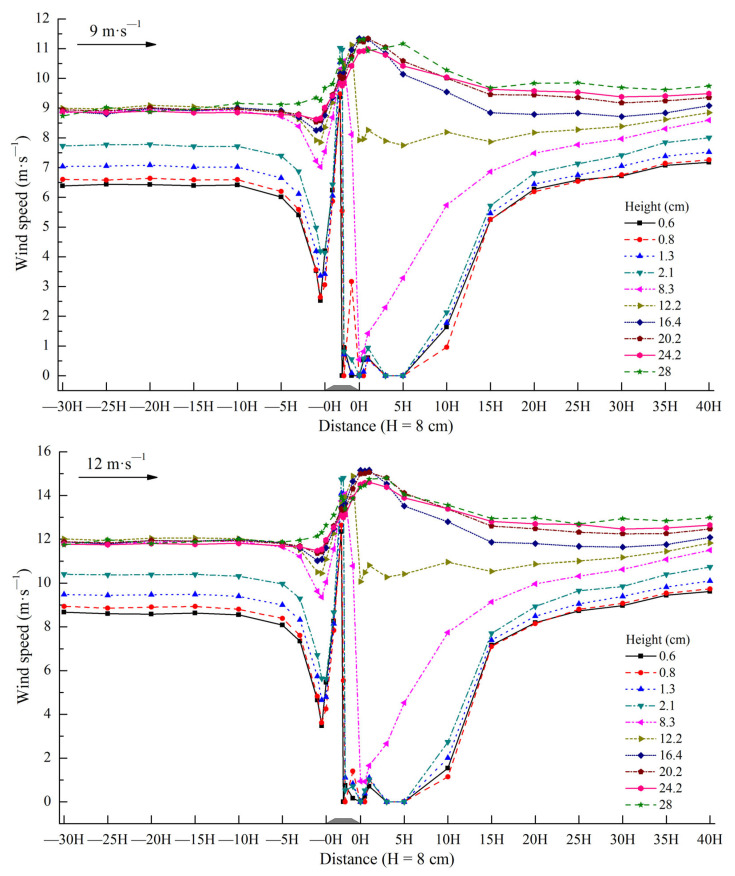

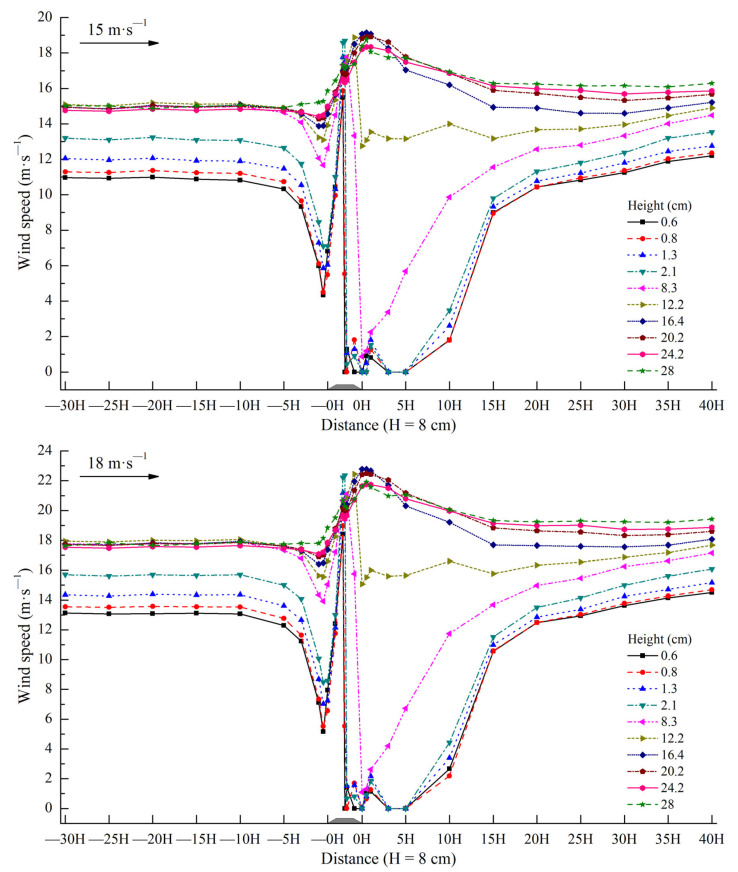
Wind speed of each observation position on the subgrade and its windward and leeward directions.

From the previously discussed wind tunnel experiment, the wind speed of each observation position on the subgrade surface and its windward and leeward sides are shown in Figure 11. A wind speed at the lower five heights (0.6, 0.8, 1.3, 2.1, and 8.3 cm) began to significantly decrease from −10H distance in the windward direction of the subgrade. The airflow speed was at a minimum at −0.5H of the windward direction and significantly increased thereafter with a climbing subgrade windward slope. The maximum appeared at the windward slope shoulder and decreased rapidly thereafter. Within the range of 0H at the leeward slope foot of the subgrade and 5H distance of the leeward direction, the wind speed dropped to a minimum (zero or close to zero) and increased significantly thereafter. The wind speed was basically restored at the 20H distance of the leeward direction. The wind speed at the upper five heights (12.2, 16.4, 20.2, 24.2, and 28 cm) started at a certain decrease from the −5H distance in the windward direction of the subgrade and gradually increased thereafter. The wind speed reached the maximum, maintained a relatively high value between the top of the subgrade and 1H distance of the downwind direction, and gradually decreased thereafter. It was basically restored at the 15H distance of the leeward direction. The variation range of the wind speed at the upper five heights was lower than that of the wind speed at the lower five heights on the subgrade surface and its windward and leeward directions. Evidently, the location at the fifth height of 8.3 cm was the bound, the subgrade influenced the range, and the extent of the wind speed ≤8.3 cm height was greater than that of the wind speed >8.3 cm height.

**Figure 12:**
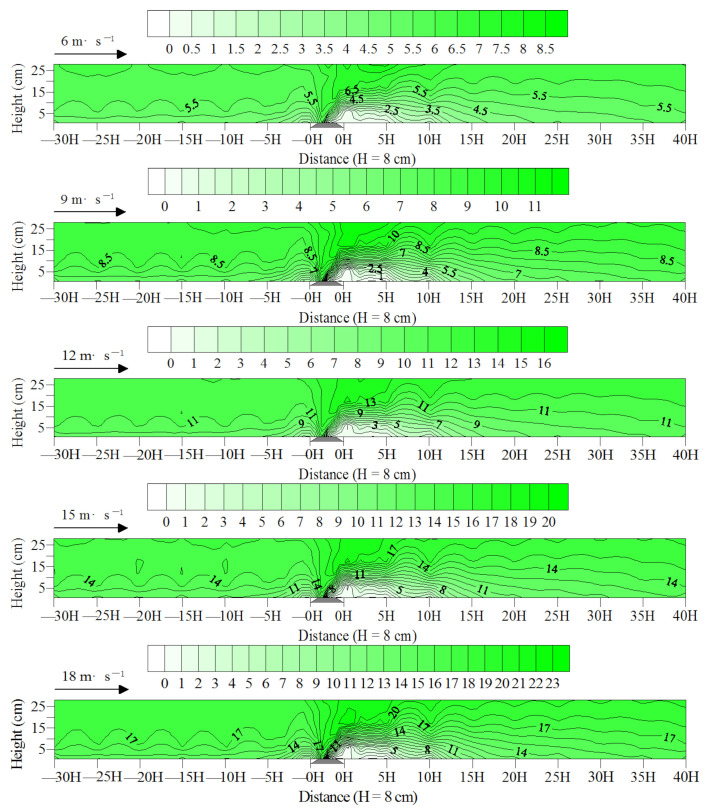
Wind flow field on the subgrade and its windward and leeward directions.

The wind flow field diagram of the subgrade surface and its windward and leeward directions is shown in Figure 12. When the wind-blown sand flow ran near the windward subgrade slope foot, substantial energy was consumed because the air flow was blocked, resulting in a decrease in the wind speed and forming a wind speed deceleration zone within the distance of −5H at the windward direction of the subgrade. The airflow gathered and accelerated thereafter with the uplift of the windward slope, forming a wind speed acceleration zone on the top of the subgrade, particularly on the windward slope shoulder. When the wind-blown sand flow crossed the slope top, it decelerated and settled due to the vortex movement, forming a wind speed deceleration zone from the subgrade top center to 20H of the leeward direction. Between the two wind speed deceleration zones, the decline range and extent of wind speed on the near surface of the latter were greater than those of the former. This finding showed that the subgrade influenced the wind speed of the leeward direction more significantly than that of the windward direction.

## 4. Discussion

**Figure 13:**
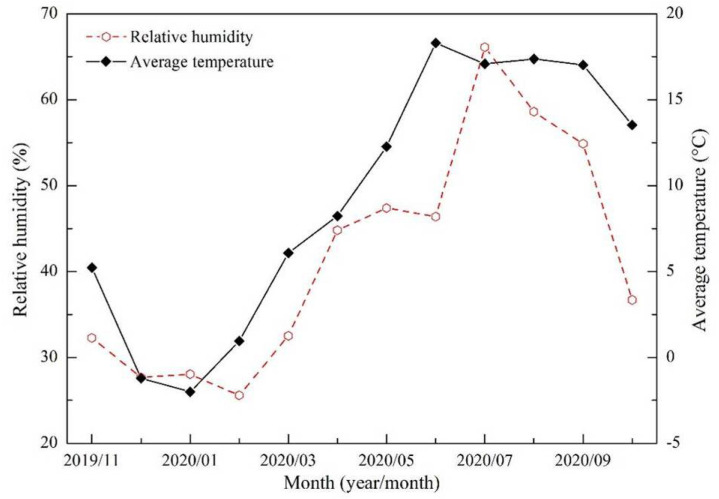
Monthly variations in the relative humidity and average temperature in the Shannan wide valley of the Sichuan–Tibet Railway.

The annual average precipitation in the Shannan wide valley of the Sichuan–Tibet Railway is 324–430 mm, which belongs to the semiarid monsoon climate of the temperate zone of the plateau. Precipitation only accounts for 10–20% of the annual total in the winter and spring. Therefore, the air is dry (Figure 13), and the water content in the sand layer of the ground surface is low. Meanwhile, the average wind speed and the frequency of sand-moving wind are high (Figure 6), and the dry and windy seasons are superimposed simultaneously, thereby further enhancing the wind-blown sand dynamic of the locale. These results can be confirmed by the calculation results of the monthly DP and Q (Figure 8), which are also similar to the existing related research results [14]. The dunes/sandy land in the Yarlung Zangbo River valley present wind-blown sand geomorphology superimposed on the fluvial landform [32,33,34]. Thus, in addition to the effect of wind, the process of wind-blown sand activity in the Shannan wide valley is also affected by the river flow action. The main features are as follows. Under the influence of the local climate, the winter half year (cold season) is dry and windy. The ground is dry and loose with abundant sand source, and vegetation is dry with low coverage. Moreover, the Yarlung Zangbo River is characterized by a decrease in the water level and weak river flow action in the dry season. The majority of the riverbed, river island, and river floodplain are exposed. In particular, the large sandy lands at the bottom of the valley are exposed, thereby providing rich material sources for wind-blown sand activities. In March, which is at the end of the cold season and the beginning of the warm season, the fluvial sediments become loose and broken from freezing to thawing. The loose sand materials are blown by the strong and dry wind, forming wind-blown sand flow, which are transported to the riverbank and valley slope and cause erosion or accumulation when they meet the blocking of the railway subgrade, thereby causing hazard. However, the summer half year (warm season) is in the same period as the rainy season, with concentrated precipitation and decreasing wind speed. The Yarlung Zangbo River is in the high-water period, the water level rises and submerges the sandy land at the bottom of the valley, and soil moisture increases. The surface vegetation recovers, and the coverage increases. The wind-blown sand dynamic and extent of the sand hazard decrease. Under the combined action of wind and Yarlung Zangbo River flow, the wind-blown sand dynamic is further enhanced in the winter half year and weakened in the summer half year, thereby resulting in the unique characteristics of wind-blown sand hazards in the alpine valley. Therefore, sand hazard in the Shannan wide valley of the Sichuan–Tibet Railway mainly occurs in the spring, specifically in March, sand hazard is severe. From a time point view, the critical period for the prevention and control of wind-blown sand hazards in the railway is the spring.

Wind is not only an important factor affecting the geomorphological evolution in sandy areas [35,36,37] but also the source power causing sand damage [38,39,40]. According to the classification standard of wind energy (Fryberger and Dean, 1979), the sand DP of the Shannan wide valley of the Sichuan–Tibet Railway indicates a low-wind-energy environment (≤200 VU), and the RDP/DP belongs to an intermediate ratio (0.3–0.8) and a closed to low ratio (≤0.3). These results demonstrate that the wind direction is dispersed, and the direction of wind-blown sand transport to the railway is multiple. When wind-blown sand flow runs near the railway, the subgrade will change its structural form and energy distribution [41]. Meanwhile, the subgrade will also affect the transport, accumulation, and erosion process of wind-blown sand flow near the surface [42]. In general, the influence increases with the increase in the subgrade volume. The wind speed deceleration zones within the distance of −5H in the windward direction of the subgrade and the subgrade top center to 20H of the leeward direction will cause sand accumulation. The wind speed accumulation zone is at the subgrade windward slope shoulder, which will cause wind-blown sand flow erosion. Therefore, sand accumulation must be controlled, given that these materials can trigger wind-blown sand flow erosion in the wind speed increased region at the subgrade windward slope shoulder. The key point for the prevention and control of wind-blown sand hazards is to establish various sand control measures, such as sand blocking and sand fixing at the periphery to prevent sand material accumulation on both sides of the subgrade. In the wind speed acceleration zone at the subgrade windward slope shoulder, the key point for the prevention and control of wind-blown sand hazards is to adopt gravel wrapping slopes and widening measures to prevent wind-blown sand flow erosion of subgrade embankment. In this wind tunnel experiment, the changes in the wind speed on the subgrade top and its windward and leeward directions are similar to relevant research results [43,44,45].

**Figure 14:**
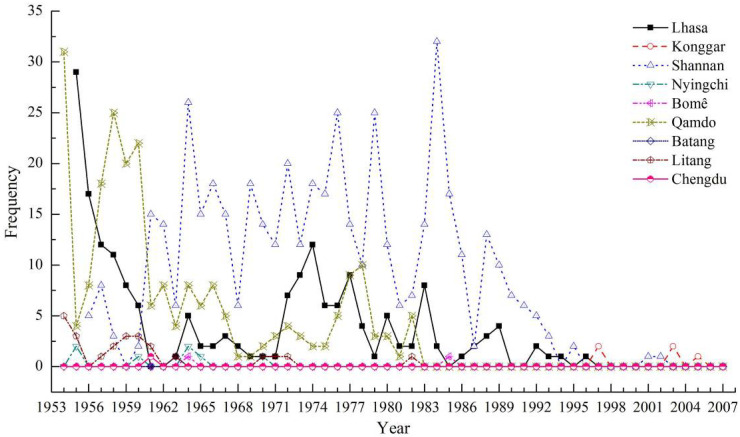
Interannual variation of dust weather at nine stations along the Sichuan–Tibet Railway.

According to the pollution degree to air quality and the harm degree to human health, dust weather is generally divided into three types, namely sandstorm, blowing sand and floating dust. Among them, the most severe is the sandstorm weather, its air horizontal visibility is less than 1.0 km. The second is the blowing sand weather, its air horizontal visibility is greater than or equal to 1.0 km and to less than 10.0 km. The third is the floating dust weather, its air horizontal visibility is greater than or equal to 10.0 km. Using “China strong dust storm sequence and its supporting data set” of China Meteorological Data Network (http://www.nmic.cn/site/index.html, accessed on 26 June 2022), the dust weather records of nine observation stations along the Sichuan–Tibet Railway were selected, the nine stations are Lhasa (29.67° N, 91.13° E, H: 3649 m, records started in January 1955), Konggar (29.30° N, 90.98° E, H: 3555 m, records started in January 1991), Shannan (29.25° N, 91.77° E, H: 3552 m, records started in September 1956), Nyingchi (29.67° N, 94.33° E, H: 2992 m, records started in January 1954), Bomê (29.87° N, 95.77° E, H: 2736 m, records started in January 1955), Qamdo (31.15° N, 97.17° E, H: 3306 m, records started in January 1954), Batang (30.00° N, 99.10° E, H: 2589 m, records started in January 1954), Litang (29.91° N, 100.27° E, H: 3949 m, records started in January 1954), Chengdu (30.67° N, 104.02° E, H: 506 m, records started in January 1954). The interannual variation of dust weather is shown in Figure 14. From the perspective of time period, the frequency of dust weather was high before the 1990s, and the frequency of dust weather decreased after the 1990s. From the perspective of location, the dust weather of Shannan station was the most severe, followed by the Qamdo station, the Lhasa station takes the third place, and the other stations rarely appear dust weather. During the observation period (1954–2007), the average annual frequency of dust weather at the nine stations along the Sichuan–Tibet Railway was 3.57, 0.29, 8.98, 0.13, 0.04, 4.20, 0.02, 0.44 and 0.02, respectively. The frequency of dust weather along the railway generally showed a decreasing trend.

**Figure 15:**
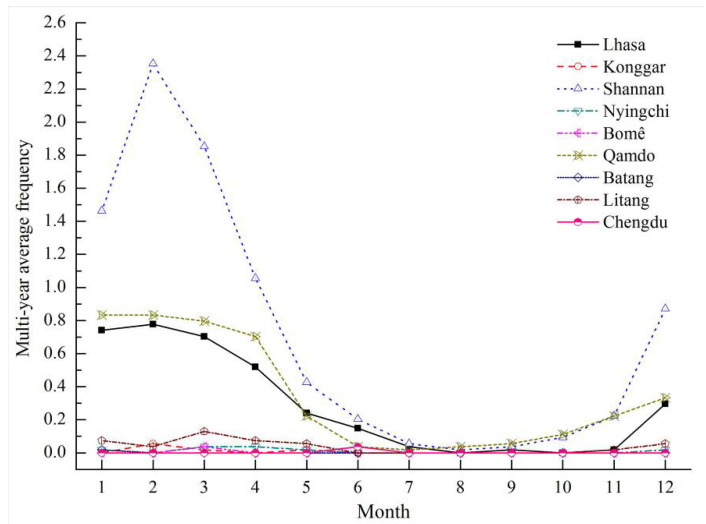
Monthly variation of multi-year average of dust weather frequency at nine stations along the Sichuan–Tibet Railway.

The monthly variation of multi-year average of dust weather frequency at nine stations along the Sichuan–Tibet Railway is shown in Figure 15. From the perspective of time, the frequency of dust weather is high in winter and spring, and low in summer and autumn. From the perspective of location, the dust weather of Shannan station was the most severe, followed by the Qamdo station, the Lhasa station takes the third place. Especially in February, the monthly frequencies of multi-year average of dust weather in Shannan, Qamdo and Lhasa are 2.35, 0.83 and 0.78, respectively. It can be seen that the dust weather along the Sichuan–Tibet Railway is mainly distributed in the three sections of Shannan, Qamdo and Lhasa from the perspective of space, and mainly occurs in winter and spring from the perspective of time. Especially in February, the dust weather overlaps with strong winds and dryness synchronously, which further polluting the air quality and aggravating the blown sand hazards along the Sichuan–Tibet Railway and thus threat to human health.

Based on the aforementioned experimental results and analysis, the prevention and control measures for wind-blown sand hazards in the Shannan wide valley of the Sichuan–Tibet Railway are as follows: sand-blocking and -fixing measures are the main measures. Sand-blocking and -fixing belts should be arranged on the outer and inner fringes of both sides of the subgrade, respectively. The width of belts in the windward direction of the prevailing wind should be greater than that of belts in the leeward direction of the prevailing wind. The large areas of mobile sand dunes far from the railway should be fixed with checkerboard sand barriers [46]. In the wind speed acceleration zone at the subgrade windward slope shoulder, gravel wrapping slopes and widening measures should be adopted to prevent wind-blown sand flow erosion of subgrade embankment.

## 5. Conclusions

The sand-moving wind of the Shannan wide valley of the Sichuan–Tibet Railway was chiefly derived from the ENE direction. The wind speed, the frequency of sand-moving wind, the DP, and the Q were higher in the spring but lower in other seasons. The annual resultant sand transport direction was WSW. Influenced by the flow action of the Yarlung Zangbo River, the dynamic of the wind-blown sand flow was further enhanced in the spring. Therefore, sand hazard mainly occurred in the spring. Particularly in March, which is the end of the cold season and the beginning of the warm season, the sand hazard was severe. Hence, the critical period for the prevention and control of wind-blown sand hazards is the spring.

The railway subgrade evidently changed the wind velocity, the wind-blown sand flow field, and conditions of transport and accumulation. Within the distance of −5H in the windward direction and the subgrade top center to 20H of the leeward direction, a wind speed deceleration zone was formed, resulting in sand particle sediment. Therefore, the key point for the prevention and control of wind-blown sand hazards is to establish sand-blocking and -fixing measures at the periphery to prevent sand material accumulation on both sides of the subgrade. A wind speed acceleration zone appeared on the subgrade windward slope shoulder, resulting in wind-blown sand flow erosion. Thus, the key point for the prevention and control of wind-blown sand hazards is to adopt gravel wrapping slopes and widening measures to prevent wind-blown sand flow erosion of subgrade embankment. The results of this investigation not only provide a scientific basis for the prevention and control of wind-blown sand hazards in the Sichuan–Tibet Railway but also have theoretical and practical significance for the prevention and control of wind-blown sand hazards in railway subgrades in other sandy areas.

## Figures and Tables

**Table 1:** Monthly variation in the resultant sand transport direction in the Shannan wide valley of the Sichuan–Tibet Railway.

Month (Year/Month)	RDD (°)	Direction	RA (°)	Direction
2019/11	105.83	ESE	105.82	ESE
2019/12	96.88	E	95.97	E
2020/01	122.97	ESE	127.58	SE
2020/02	179.06	S	179.33	S
2020/03	126.56	SE	125.75	SE
2020/04	233.89	SW	230.40	SW
2020/05	262.36	W	262.16	W
2020/06	256.18	WSW	255.62	WSW
2020/07	265.94	W	266.37	W
2020/08	260.52	W	259.60	W
2020/09	257.95	WSW	257.78	WSW
2020/10	270.97	W	271.11	W

**Table 2:** Q of each wind speed class in the Shannan wide valley of the Sichuan–Tibet Railway.

Wind speed range (m·s^−1^)	5–6	6–7	7–8	8–9	9–10	10–11	11–12	12–13	13–14
November 2019 to October 2020 Q (kg·m^−1^·a^−1^)	2.98	21.05	28.72	19.39	10.41	4.79	3.51	0.91	0.47

## Data Availability

Not applicable.
